# Biomedical research competencies for osteopathic medical students

**DOI:** 10.1186/1750-4732-3-10

**Published:** 2009-10-13

**Authors:** des Anges Cruser, Bruce Dubin, Sarah K Brown, Lori L Bakken, John C Licciardone, Alan L Podawiltz, Robert J Bulik

**Affiliations:** 1 Department of Social and Behavioral Sciences, School of Public Health, University of North Texas Health Science Center, 3500 Camp Bowie Blvd., Fort Worth TX, USA; 2 Texas College of Osteopathic Medicine, University of North Texas Health Science Center, Fort Worth TX, USA; 3 Mental Sciences Institute, University of North Texas Health Science Center, Fort Worth TX, USA; 4 Department of Medicine, School of Medicine and Public Health, University of Wisconsin, Madison WI, USA; 5 Department of Family Medicine and the Office of Educational Development, University of Texas Medical Branch at Galveston TX, USA

## Abstract

**Background:**

Without systematic exposure to biomedical research concepts or applications, osteopathic medical students may be generally under-prepared to efficiently consume and effectively apply research and evidence-based medicine information in patient care. The academic literature suggests that although medical residents are increasingly expected to conduct research in their post graduate training specialties, they generally have limited understanding of research concepts.

With grant support from the National Center for Complementary and Alternative Medicine, and a grant from the Osteopathic Heritage Foundation, the University of North Texas Health Science Center (UNTHSC) is incorporating research education in the osteopathic medical school curriculum. The first phase of this research education project involved a baseline assessment of students' understanding of targeted research concepts. This paper reports the results of that assessment and discusses implications for research education during medical school.

**Methods:**

Using a novel set of research competencies supported by the literature as needed for understanding research information, we created a questionnaire to measure students' confidence and understanding of selected research concepts. Three matriculating medical school classes completed the on-line questionnaire. Data were analyzed for differences between groups using analysis of variance and t-tests. Correlation coefficients were computed for the confidence and applied understanding measures. We performed a principle component factor analysis of the confidence items, and used multiple regression analyses to explore how confidence might be related to the applied understanding.

**Results:**

Of 496 total incoming, first, and second year medical students, 354 (71.4%) completed the questionnaire. Incoming students expressed significantly more confidence than first or second year students *(F = 7.198, df = 2, 351, P = 0.001) *in their ability to understand the research concepts. Factor analyses of the confidence items yielded conceptually coherent groupings. Regression analysis confirmed a relationship between confidence and applied understanding referred to as knowledge. Confidence scores were important in explaining variability in knowledge scores of the respondents.

**Conclusion:**

Medical students with limited understanding of research concepts may struggle to understand the medical literature. Assessing medical students' confidence to understand and objectively measured ability to interpret basic research concepts can be used to incorporate competency based research material into the existing curriculum.

## Introduction

The immutable relationship between research and medicine shapes the contours of the medical education terrain. Evidence-based medicine (EBM) principles populate the landscape of this terrain as protégés of that relationship. As medical students cover this medical education terrain, they metaphorically run the rapids between the knowledge generated by scientific research upstream and the guidelines of clinical practice downstream. A few research-motivated students may explore the research aspects of this landscape, taking advantage of various research related opportunities. The vast majority of students however, cover the medical education curricular terrain with very limited exposure to the language and concepts of research that nurture EBM.

With the support of a Research Education Project Partnership (R25) grant from the National Center for Complementary and Alternative Medicine (NCCAM), the University of North Texas Health Science Center (UNTHSC) is integrating information on the language and culture of biomedical research into the medical school curriculum. With funding from the Osteopathic Heritage Foundation we will offer this research education model to other interested osteopathic medical schools. This research education curriculum focuses on equipping all medical students with basic research competencies, and supports research education activities in manual medicine and selected Complementary and Alternative Medicine (CAM) therapies. A key component of this research education curriculum was a baseline measurement of students' confidence in interpreting, and ability to apply basic research concepts as well as their attitudes toward research. This paper reports on one aspect of that baseline assessment taken in May 2008; that is the students' self-evaluated confidence that they could interpret basic research concepts. A separate paper will provide detailed information on the knowledge questions, from both the baseline and the post-curriculum assessment taken in May 2009.

## Background

To strengthen research education for a variety of pre-doctoral health sciences students, NCCAM has awarded approximately 26 four-year R25 grants since fiscal year 2000, to academic health science institutions in the United States. The first round of R25 grants focused on developing CAM curricula for conventional health care professionals [1]. The second round of R25 grants was awarded to CAM teaching institutions eligible according to the NCCAM definition. Osteopathic medical schools qualified for these grants because the core curriculum requires training in manual manipulation, defined as a CAM modality. For an R25 grant, the institution must establish a consortium partnership with a conventional institution as a required research intensive partner. NCCAM objectives for these R25 grants focus on increasing the research content in the pre-professional (undergraduate/doctoral) curriculum to enhance practitioners' abilities to critically evaluate the biomedical literature, and improve their capacities to participate in clinical research with a goal of stimulating some students to seek advanced research training.

It is generally accepted that biomedical research competencies for clinical researchers develop along a continuum that ideally begins with exposure of pre-professional students to the language and culture of research, and extends through post-doctoral training to the independent conduct of multi-center clinical trials. This suggests that a multi-tiered competency model might be useful to differentiate among basic, intermediate, and advanced research proficiencies.

**Figure 1:**
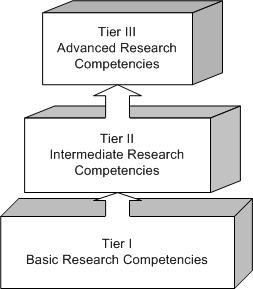
**Biomedical Research Competencies Three-Tiered Education Model**.

The UNTHSC research education program uses the novel three-tiered model illustrated in Figure 1. In this model, Tier I competencies are those that characterize all students and practitioners who are proficient professional consumers of biomedical research literature. Tier I is the primary focus of this R25 grant.

Tier II research competencies comprise transformative cognitive, affective, and psychomotor skills an individual needs to apply in conducting mentored research, or for meaningful (e.g. publishing, presenting scholarly work, conducting a sub-study) participation in on-going research. A Tier II student, for example, would be training in a dual-degree master's of science track, or producing scholarly work from an elective rotation in on-going research. Tier II faculty would have a master's degree, and would be able to confidently teach Tier I competencies.

Tier III students would be in a dual degree doctoral program, or, armed with a pre-doctoral master's degree in a research field, they may be in a post-doctoral research fellowship. Tier III faculty are those with a master's degree and additional training, or with a Ph.D. or post-doc research training. Tier III faculty are qualified to teach and mentor Tier II students, and to mentor Tier III students.

The NIH makes a distinction between *research training *and *research education *in its funding strategies by focusing certain types of awards such as the K23 and T32 on training in the conduct of research, and the R25 and K30 on research education such as curriculum development or enhancement. It may seem an artificial distinction, but can be differentiated by thinking of *research training *as a program for mastering skills to conduct research, and *research education *as a program for developing proficiencies as a professional consumer of research, critically evaluating and using research information in clinical practice.

In Tier I, we differentiate *research education *from *research training*, and we define knowledge (through education) as the range of one's information or understanding, and competence (through practice) as having a capacity to function in a particular way. Competencies in the available *research training *literature are more suited to those skills needed at Tiers II and III performance levels. Specifically, we have evaluated the medical students for their understanding of targeted research concepts at a Tier I level.

There are three broad aspects of contemporary clinical research training programs that provide important landscape materials for this Tier I research education initiative. One is self-efficacy theory, and the others are biostatistics and evidence-based medicine (EBM).

### Self-Efficacy Theory

Self-efficacy theory is a theoretical construct that helps to understand how belief in one's capabilities to perform a particular behavior aimed at a desired result may influence one's performance in the area of interest [2]. Extensive information is available in self-efficacy theory for the interested reader, but for the purpose of this paper, it is important to know that self-efficacy is based on a belief that to perform any task successfully, one needs both confidence and competence in the relevant skill set. For example, career self-efficacy would be a factor explaining why someone selects a particular career and performs well in that career in spite of possible obstacles to success. It is generally believed that the earlier exposure one has to any career, the more likely the individual will choose to engage in that career [3-5].

The literature in research training provides a well developed construct for research self-efficacy (RSE). RSE can be measured, and can help us understand why one might choose, persist in, and achieve confidence in a research career. RSE is a major factor in whether one chooses to participate in research, or to pursue a research career despite obstacles. While RSE is clearly not the only predictor of career choice or performance in research, it does provide a useful construct to begin to understand how confidence, and knowledge or competence, are related to career choice and performance. Up to now, this construct has not been used to assess biomedical research confidence or knowledge among non-research motivated pre-doctoral osteopathic medical students.

RSE has been measured among post-doctoral physician research trainees using several different inventories including the 51-item Research Self-Efficacy Scale (RSES) [6], and the 33-item Self-Efficacy in Research Measure (SERM) [7]. Reviews of these instruments [8] and of the empirical evidence from RSE studies suggest that each professional environment and knowledge domain should be assessed with items that most closely represent the unique features of that profession. While many different dimensions can be measured with RSE inventories, there is general consensus that the most relevant domains would be behavior specific, and include decision making, task management, public speaking, data analysis, research integration, and data collection [9]. These domains reside more appropriately in Tiers II and III competencies in our model. Thus we speculate that for medical students, research self-efficacy is different, and would be comprised of different domains. These domains might include defining research terms, discussing methods and validity, or understanding human subjects protection issues. There was no single existing RSE inventory that tapped the domains we identified in Tier I as competencies for a proficient professional consumer of biomedical research information.

One other aspect of RSE important to our research education project is the relationship between proficiency in a task and confidence to perform that task [5,10]. One could speculate intuitively that knowing how to perform a task or achieve a desired outcome would increase one's confidence and satisfaction, but the value we found in the RSE construct was the way it helps to explain the choice of a research career, and one's dedication and success in that career. Research tasks per se, being more relevant to Tiers II and III competencies, require skills in performing research tasks, whereas knowledge, one's range of information or understanding, provides a more precise way of defining Tier I research competencies for all medical students.

Additionally we were interested in whether students' self-rated confidence to interpret a given research concept was related to their objectively tested ability to apply that concept. Confidence and competence are two factors known to predict performance, and we do not know whether medical students have confidence equal to their actual ability to understand and apply research concepts. Assessing students' confidence and competence can provide valuable information for creating appropriately targeted curricular content and methods to teach research literacy. Knowing the students' confidence and their objectively measured competence can help to ensure that instructional methods do not over or underestimate students' readiness to achieve research literacy. Findings of RSE studies have been useful in creating innovative curricula for a variety of health professions students to increase their research thinking and interest [11,12]. Therefore the basic construct of research self-efficacy provided a suitable theoretical framework for our assessment of confidence and competence in Tier I research competencies.

### Biostatistics

The second broad aspect of contemporary clinical research training programs that provides important landscape materials for this Tier I research education initiative is biostatistics. Biostatistics or medical statistics are a sine-qua-non of research. In an incremental approach to research education, understanding certain biostatistics concepts is an essential Tier I research competency, but only at the level required to be a proficient professional consumer of research information. While Tiers II and III competencies require skills in advanced analytic statistics, research motivated students must take several courses or be rigorously mentored in these skills. Statistics training sufficient to be competent at a Tier II level may not be feasible for all medical students. Thus, if we agree being a proficient professional consumer of research information requires some conversancy with biostatistics, but not computational statistics, how can we teach this within the constraints of the medical education curriculum? For Tier I competencies we utilize methods that teach a basic applied understanding of biostatistics using, among others, the principles in the text "Intuitive Biostatistics" [13].

Biostatistics knowledge can be measured in many ways. For example, measurement scales are available for assessing students' attitudes toward and knowledge of biostatistics in bachelors or graduate biostatistics degree programs. The most widely used measures in published educational research, are the Survey of Attitudes toward Statistics Scale [14], the Attitudes Toward Statistics [15], and the Survey of Attitudes Toward Statistics [16]. Studies have also been published using modified versions of these questionnaires [17]; but these are of limited value to assess non-research motivated medical students' understanding of biostatistics concepts. There have also been direct measures of knowledge used to assess post-graduate trainees' understanding of medical statistics in the literature [18] which target Tiers II and III competencies, and are therefore indicative of what questions can be used to measure Tier I research and biostatistics competencies in the context of research education for medical students.

### Evidence-Based Medicine

The third broad aspect of contemporary clinical research training programs, providing important landscape materials for this Tier I research education curriculum is evidence-based medicine (EBM). EBM is the conscientious, explicit, and judicious use of current, published scientific research information. EBM has become a nearly essential component of decision-making in patient care. While it is not necessary to be conversant with all aspects of EBM to understand the issues this paper addresses, it is important to keep in mind that biomedical research concepts and EBM principles are interrelated. To apply EBM information in the real world of treatment, experts recommend using the lenses of research principles, concepts and methods. EBM should not be confused with biomedical research per se, in that there is a hierarchy of evidence with which all health care professions work that may in fact be independent of scientific or published research. The EBM paradigm is an open ended, constantly evolving system that represents an extension of field and clinical epidemiology effectively intertwined with biostatistics and the experience of the practitioner [19].

Current methods of teaching EBM can be algorithmic, and may underutilize the rich contours of the language and culture of research. EBM and research literacy together may be better than either one alone. Thus students should not learn only EBM or only research concepts. While students learn how to read the research literature, they should understand its role in EBM and utilize critical thinking to avoid adopting extremely credulous or incredulous attitudes toward research. It is critical for future health practitioners to understand that research and evidence-based information may be believable even though flawed, and also may be of value within known limitations.

## Methods

Our primary outcomes for the pre-curriculum assessment were self-rated confidence to understand Tier I biomedical research concepts (confidence), and objectively measured ability or competence to apply that understanding (referred to as "knowledge"). We developed a novel questionnaire using some items from the published literature cited above, and additional new items developed by clinical scientists and biostatisticians on the faculty at UNTHSC. The final product was a questionnaire measuring confidence with 32 statements, and knowledge with 20 multiple-choice questions requiring an interpretation of Tier I research concepts. With the approval of the institutional review board, this questionnaire was administered in May 2008, to the incoming, first, and second year classes of osteopathic medical students at UNTHSC.

### Basic Biomedical Competencies

The Tier I research competencies listed below, were developed by working backward from published competencies in the Tiers II and III domains that represent research competencies for dual degree and post-doctoral clinical research trainees and physician scientists [20,21,14,17]. Tier I research competencies are defined for this project as those that enable a student to read and critically evaluate the research literature. These competencies guided the development of the questionnaire. The students at the end of the Tier I curriculum are expected to be able to recognize different types of the most common research designs, and be familiar with these commonly used concepts in research: validity and reliability of a measurement and of research studies/designs; sensitivity and specificity of a testing method or instrument; the most commonly used types of variables; power analysis and its importance to research; essential components of a written literature review; qualities of a well written research question or hypothesis and abstract; type I and type II errors; P values, confidence intervals, odds ratios, relative risk; and the most commonly used statistical concepts and tests e.g. Chi-square, t-tests. By the end of the Tier I curriculum students are expected to know how to use a decision tree to determine if a given research report used an appropriate statistical analyses for the question and the type of data; identify what is needed to calculate/estimate sample size for a given study; distinguish between parametric and non-parametric statistics; and identify and recognize strengths and limitations of selected research designs. In the area of human subjects, they are expected to be able to identify and discuss basic ethical issues in human subjects research including the basic principles of ethical human subjects research. They should recognize differences between the FDA and DHHS research regulations, define the purpose of the Institutional Review Board; identify the essential components of a research study consent form; discuss placebo issues, and identify some of the differences between a patient and a research subject. Students present an intramural critical evaluation of a simple to minimally complex research article including an interpretation of the published study's findings, and a discussion of the "statistical assumptions" for the tests of the hypothesis, threats to internal validity, and strengths and limitations of the research design. Tier I students are also expected to be able to efficiently perform a focused literature search. They may participate in on-going human subjects or basic research, and organize and present a research poster

### Confidence

Using previously cited literature and the Tier I research competencies, we developed an initial set of 50 statements asking the students to indicate their level of confidence to understand or apply targeted research concepts. A team of clinical and research faculty reviewed six articles in research self-efficacy and the associated measurement instruments acquired from the authors. They then completed the 50-item confidence questionnaire in a group, and held three separate face-to-face group discussions of each confidence statement over the span of two weeks, facilitated by the project principal investigator (PI). Final agreement was reached on 32 statements that were subsequently endorsed by the multi-disciplinary research education committee which includes medical and biomedical sciences graduate school deans and course directors, and external expert consultants. Each confidence statement required a response on a scale of 1 to 4, with 1 representing "not at all confident", and 4 representing "completely confident".

### Applied Understanding of Research Concepts

We refer to the students' objectively measured ability to apply targeted research concepts as "knowledge," which was measured with multiple-choice questions. Every question testing the application of the research concept offered four solutions. We used questions from published research [12,18] and added novel questions developed by selected UNTHSC physician scientists and biostatistics faculty based on Tier I research competencies. Selection of final knowledge questions was guided by the understanding that medical students should focus on a primarily conceptual formulation of biostatistics or medical statistics rather than a computational formulation, although understanding of some computations was necessary. Questions were framed in case contexts or in interpretive frameworks.

Thirty multiple-choice questions were considered for inclusion by physician-scientist faculty on the research education committee both through independent review, and group discussions of each item facilitated by the project biostatistician and the project PI. A group of 12 first and second year student volunteers completed the entire questionnaire before it was deployed, providing feedback on the wording and organization of the questionnaire. Those twelve students did not re-take the questionnaire, nor were their data included in the analysis. The research education committee ultimately agreed on 20 multiple choice knowledge questions linked to the Tier I research competencies.

### Data Collection and Analysis

With the approval of the UNTHSC Institutional Review Board, we administered the questionnaire to three cohorts of students in May 2008. The survey was provided on-line through the university's secure server for the entire class to complete during the normal school day for first and second year students, proctored by the Clinical Medicine course director and project PI. Incoming students were e-mailed a link by the academic dean with instructions to complete the questionnaire before arriving at campus for orientation in June.

Students responded first to confidence items, second to knowledge questions, and last to attitude. The questionnaire was structured to require a response to each item before being able to proceed to the next item or section. Students were instructed to avoid guessing their answers to the knowledge questions. In addition to the four solutions offered, each question included a fifth "no response" (NR) choice. The reason for this was to be able to examine relationships between what the students said they knew (confidence) and what they actually reflected they knew (knowledge). First and second year students required an average of 30 minutes to complete the questionnaire. We do not have data on the length of time incoming students required to complete the questionnaire. The questionnaire could not be saved for return sessions, and had to be completed in one session.

Questionnaire results were transferred from the university's secure on-line survey platform without identifiers to an excel spreadsheet and imported into SPSS version 15 for cleaning and analysis. Analysis of the data included descriptive statistics, and analysis of variance and t-tests to determine differences between groups for confidence and knowledge. We utilized regression analysis to further explore the relationship between confidence and knowledge scores. Because the instrument is a novel one, we computed Cronbach's alpha to estimate the reliability of the items, and performed a principal components factor analysis (PCA) to determine whether confidence items clustered into coherent groupings.

## Results

Of 496 incoming (178), first (155) and second year (163) medical students, 354 (71.4%) completed the questionnaire, 25.4% in the incoming class of 2012, 40.4% in the first year class of 2011, and 34.2% in the second year class of 2010. Among this group of students 258 (72.9%) had a bachelor's degree, and 68 (19.2%) had a master's or a doctoral degree, or a post-baccalaureate certificate, with 28 students' previous education unreported. In this sample there were 154 (43.5%) females and 174 (49.2%) males (26 did not report gender). For race, 184 (52%) students reported as White, 90 (25.4%) reported Asian, 34 (9.6%) reported Hispanic, and 14 (4%) reported Black. There were 32 students reporting undeclared or other racial affiliation. The average highest MCAT score was 27.1 (SD 2.8). Table 1 displays summary statistics by class for total confidence and knowledge scores.

**Table 1:** Respondents Confidence and Knowledge by Class

**Class** **N (%)**	**Respondents** **N (%)**	**Total Confidence** **Mean (SD)**	**Questions Attempted** **Mean (SD)**	**Number Correct** **Mean (SD)**	**% Correct of Attempted** **Mean (SD)**	**% Correct of 20** **Mean (SD)**
Incoming 178 (35.9%)	90 (50.5%)	84.5 (19.2)	11.7 (6.1)	6.2 (3.6)	51.6% (20.6)	31.1% (18.1)
First Year 155 (31.3%)	143 (92.3%)	78.2 (17.7)	11.0 (6.4)	5.3 (3.4)	46.6% (21.4)	26.5% (17.0)
Second Year 163 (32.8%)	121 (74.2%)	74.7 (19.5)	12.3 (6.1)	5.7 (3.2)	47.2% (20.5)	28.4% (15.9)

Total 496 (100%)	354 (69.6%)	78.6 (19.0)	11.6 (6.2)	5.7 (3.4)	48.1% (20.9)	28.3% (17.0)

### Confidence

Cronbach's alpha reliability coefficient is 0.966 for the confidence items. Confidence items were highly intra-correlated. We computed a measure of "total confidence" ranging from no-confidence at 32, to complete confidence at 128.

Analysis of variance indicated that the three class groups differed significantly in total confidence (*F = 7.198, df = 2,351, P = 0.001*), with incoming students identified with a post-hoc Tukey Honestly Significant Difference test as significantly more confident on average than first or second year students. We compared the students by type of pre-medical school degree (graduate degree or certificate, compared to bachelors degree only) using an independent T-test, and found that those with a pre-enrollment graduate degree or certificate had a significantly higher mean confidence score of 85.4, while those with a bachelor's degree-only scored an average of 76.2 *(t = 3.6, df = 324, P < 0.001)*. There were no differences in total confidence for race or gender.

In keeping with the literature that suggests that behavior domain-specific aspects of self-efficacy should be measured using profession-relevant dimensions, we utilized a principal component factor analysis (PCA) of the confidence items to determine whether they correlated in any coherent patterns suggesting relevant domains for Tier I. An initial un-rotated non-forced component matrix produced only one factor. Using a varimax rotation, allowing for a maximization of the variance of the loadings, and forcing a 4-factor solution, we obtained the results displayed in Table 2.

**Table 2:** Research Confidence

	**FACTOR LOADINGS**
Factor 1 Defining Selected Methods (19%)	
Describe the purpose/use of a t-test.	.692
Describe assumptions for use of a statistical test.	.686
Describe the use of statistical regression analysis.	.686
Define the purpose of the chi-square statistic.	.672
Define a Type-I or Type-II error in a research study.	.671
Interpret inferential statistics.	.660
Interpret "p-values" for a given results.	.659
Identify information needed for perform a power analysis and sample size estimation.	.640
Clearly define an outcome measure.	.583
Describe the meaning of mean and standard deviation.	.481
Factor 2 Developing and Discussing Research (18%)	
Interpret descriptive statistics.	.694
Critically evaluate a medical research article.	.673
Apply the results of a study to aid in developing a differential diagnosis.	.576
Present research findings in a poster.	.574
Determine the sensitivity and specificity of a test.	.562
Select an appropriate statistical method for a research questions.^1^	.560
Interpret odds ratios.^1^	.551
Discuss methodological cautions in interpreting research findings of a given study.	.538
Correctly recognize different types of research designs.	.534
Design your own research study.	.529
Present a critical review of the strengths and limitations of a research study.	.521
Correctly identify different types of variables.	.514
Correctly identify major threats to internal and external validity for a given research design.^2^	.483
Factor 3 Developing Clinical Research Proposals (15%)	
Identify the essential elements of informed consent for research participation.	.794
Discuss current ethical issues related to the use of placebos in clinical trials.	.752
Explain the purpose of an Institutional Review Board proposal.	.735
Discuss the reasons for human subjects protection regulations in research.	.635
Correctly identify the essential parts of a research proposal.	.546
Efficiently perform a topic-focused literature search.^3^	.444
Factor 4 Evaluating Research Integrity (11%)	
Provide a working definition of validity.	.742
Provide a working definition of reliability.	.702
Define the concept of variability (systematic and non-systematic).	.616

**TOTAL VARIANCE EXPLAINED**	**63%**

^1 ^These two items loaded approximately equally in factors 2 and 1

^2 ^This item loaded approximately equally in factors 2 and 3

^3 ^This item loaded approximately equally in factors 2, 3, and 4

This four-factor solution offers a reasonable preliminary set of sub-scales, with four items that cross-load with some arguable logic. A 3-factor solution shifted some of the items that cross-loaded in the 4-factor solution to other sub-scales, but also had additional cross-loaded items, and a less conceptually elegant relationship among the items in each factor.

We have labeled the four factors as 1) Defining Selected Methods, 2) Developing and Discussing Research, 3) Developing Clinical Research Proposals, and 4) Evaluating Research Integrity. Four items loaded about equally on more than one sub-scale, indicating a strong inter-correlation with several items.

### Applied Understanding of Research Concepts

The complete baseline assessment included 20 multiple-choice questions targeting specific knowledge of research methods and techniques. While not every confidence statement had an associated knowledge question, there was significant overlap. A separate paper will provide the details of the knowledge questions and include analysis of the pre and post curriculum results. For this paper however, we believe it is useful to provide the reader with a general overview of how the students' baseline (pre-curriculum) performance on knowledge questions is related to their self-rated confidence in understanding these targeted basic Tier I research concepts.

For all 20 questions, the mean (SD) number of correct answers was 5.7 (SD = 3.4); and the mode was seven. Eight students correctly answered 65% or more of the questions. This corresponds to an average score of 28.5% for all respondents.

We included a no-response (NR) choice for every question to discourage guessing and encourage the student to answer only those questions they felt confident to answer. Students were instructed to use the NR option if they felt they could only guess at the answer. This provided us with a buffer between random correct answers from guessing, and deliberate attempts on the part of the student to answer only the questions they felt confident to answer.

On average all students attempted 12 of the 20 questions. Forty-four students (12.4%) attempted all 20 questions; 38% attempted 15 or more and only 3% did not attempt any. No student correctly answered all questions they attempted. On average, all respondents correctly answered 48% of the questions they attempted, and 28% of all 20 questions. The mean number of times NR was selected was 8.4 (SD = 6.2), with a median of 8.

To examine the relationship between confidence and knowledge, we used the total number of correct answers, and the number of questions attempted (i.e. not a no-response choice) to compute a Pearson's correlation coefficient. Total confidence was moderately related to the number of correct answers (*r = 0.448, P < 0.001)*, and to the number of questions attempted (*r = .0368, P < 0.001)*.

Because measuring confidence alone provides little useful information for crafting a curriculum in research, and because we have, at baseline, a significant correlation between confidence and knowledge, we used regression models to further explore that relationship. Table 3 displays the results of the multiple regression models we used to explore the extent to which pre-enrollment characteristics and confidence explain knowledge. The model that most strongly explained knowledge *(R*^2 ^* = 0.242) *was the model including the measure of total confidence.

**Table 3:** Regression Analysis Estimating Knowledge

	**Model 1**		**Model 2**	
	***R*^2 ^= 0.058**		***R*^2 ^= 0.242**	
	***β***	***P***	***β***	***P***
Medical School Experience				
Completed Year 1	-0.581	0.231	-0.079.	0.857
Completed Year 2	-0.182	0.716	0.628	0.173
Post-Bachelors Education	1.259	0.006	0.526	0.212
Highest MCAT Score	0.238	<0.001	0.271	<0.001
Overall Confidence			0.080	<0.001

Available information about students in this sample that might explain knowledge, includes years in medical school, post-bachelor pre-medical school education, MCAT scores, and confidence for research concepts. For Model 1, examining the ability of selected information about students' educational background to explain the knowledge scores, indicates that education beyond a bachelors degree (*P *= 0.006) and MCAT score (*P *< 0.001) are associated with more correct answers. In Model 2, the addition of total confidence to the model greatly increases the ability of the model to explain knowledge scores, and confidence becomes the most important variable explaining the variance in the total number of correct answers to knowledge questions (*P *< 0.001).

### Strengths and Limitations

This study utilized validated concepts within the theoretical construct of research self-efficacy. The questionnaire included published and novel items in measuring confidence and knowledge. However, unlike other tests of statistics knowledge, students were given the option of selecting a "no-response" (NR) choice instead of guessing the answer. Although one might speculate that students would use the NR choice simply to avoid having to read and consider the questions, the relationship between questions attempted and percent of all questions answered correctly suggests that the students used the NR option judiciously. A second paper will provide analysis of the knowledge questions in more detail.

This pre-curriculum assessment served a practical purpose of testing the notion that medical students might be over-confident, thus over-estimating their research competence, which it appears they did only slightly. While this approach to measuring osteopathic medical students' understanding of research concepts has been used by others, this study is limited to this group of UNTHSC medical students and may not be generalizable to other medical students. We must also acknowledge that although every effort was made to refine knowledge questions to avoid any confusion in the response choices, human error is a possible confounding variable for the validity of this novel instrument. Instrument validity and reliability will be further evaluated with larger and more diverse samples and repeated measures.

## Discussion

### Confidence

While the four factors of the confidence items are arguably domain specific, there is some overlap, suggesting that Tier I research competencies cross multiple domains. This is evident in the four items that loaded about equally in two or three factors. Two of the items in "Developing and Discussing Research" (*select an appropriate statistical method for a research questions*, and *interpret odds ratios*) might be equally associated with "Defining Selected Methods," and a third item "*correctly identify major threats to internal and external validity for a given research design*" might be equally important to "Developing Clinical Research Proposals." The fourth cross-loaded item "*efficiently perform a topic-focused literature search*" loaded approximately equally on three domains. We included that item in "Developing Clinical Research Proposals" although it might also be associated with "Developing and Discussing Research" or "Evaluating Research Integrity."

Being able to perform a topic-focused literature search may be as important to understanding research as it is to constructing research. We do not expect all medical students to be able to competently develop a research proposal, but we do expect them to recognize quality in the formulation of a research question, and the essential parts of a research paper according to current convention [22,23].

We observed that the incoming class expressed significantly greater confidence in their ability to interpret or apply targeted research concepts. At this college of osteopathic medicine there are increasing annual proportions of entering students who have completed a pre-enrollment master's degree and thus may have had more exposure to these Tier I competencies than those in the previous classes. This is a possible explanation for their higher self-rated confidence that may be tested with future years of data.

Determining how behavior domains for understanding research differ from those required for conducting research, would require the use of additional separate validated inventories, and repeated measures. It will also be important to monitor whether student participation in research or scholarly activities increases over the coming years, possibly as a result of achieving foundational skills in understanding research concepts. Confirmatory factor analysis with our forthcoming post-curriculum data will also help to refine these domains.

### Applied Understanding

Students' knowledge scores indicate that they have much to learn about research concepts and terminology. We would not expect medical students to be able to answer all of these knowledge questions correctly. Post-baccalaureate pre-medical school education apparently gives some students an edge in interpreting research concepts, but the overall knowledge of research concepts is still limited in this sample of osteopathic medical students. Although the correlation is only moderate between total confidence and overall knowledge in this group, confidence is a key concept in explaining the variance in knowledge scores. In fact, it may prove useful in estimating knowledge related to research competencies, more so than traditional measures such as prior degrees or MCAT scores.

### Description of the Curriculum

**Figure 2:**
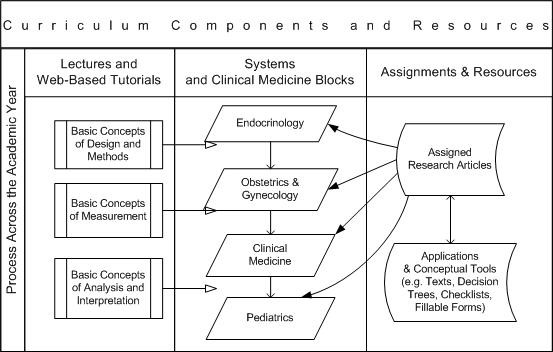
**Example of an Integrated Curriculum in Tier I Biomedical Research Competencies**.

To better understand how the targeted Tier I research competencies are addressed in the novel curriculum being used at UNTHSC, we provide a diagram in Figure 2.

The model uses an iterative process across the academic year. First the students are exposed to traditional lectures as illustrated in the first column, and provided with on-line lectures and tutorials. Selected research articles topically correspond with system blocks or clinical medicine topics. This article may be about Legionnaires Disease for example, or perinatal depression, pediatric oncology, or influenza. Web-based application tools and conceptual tools are provided to guide the students' critical evaluation of the research information. They are not expected to understand all the concepts in the beginning of the course. Research concepts and language are introduced in increasingly sophisticated stages, with each article selected to reflect that stage. Small groups of students present grand rounds style critical reviews of the assigned articles to their classmates and faculty using a critical evaluation guide and slide templates. Faculty reviewers provide feedback to the students on their presentations. Exam questions on the research concepts covered in each article represent 10% of the student's grade in the clinical medicine course.

## Conclusion

In a recent discussion of the past 14 years of EBM, Milos Jenicek, M.D., Ph.D. encourages us to appreciate and develop new and better ways of thinking about evidence in patient care. If we want our students to appreciate scientific information we should consider equipping them with higher order skills for interpreting, evaluating, and inferring from research information, what it is they understand, believe, and intend to use in the practice of medicine. The curriculum for this research education project attempts to address the three questions Jenicek poses: (1) How can we teach lateral thinking skills for understanding research in the context of clinical practice? (2) Is there potential evidence-base value in other research such as qualitative social science or public health domains? (3) What, when, and how should we teach medical students in areas of epistemology, logic, critical thinking vis-ΰ-vis research and EBM? [19]

Despite a nationally recognized need for clinical researchers in all fields, and requirements for scholarly activity in residency training programs, biomedical research competencies are under-represented in the osteopathic medical school curricula. This may be partly because there is no consensus regarding the content or level of biomedical research competencies students should achieve before completing their undergraduate medical education. Although efforts and recommendations have been made in the Osteopathic profession by policy committees and operational groups, there is currently no official position taken by the American Osteopathic Association or the Association of Colleges of Osteopathic Medicine regarding required research competency components in the medical school curriculum.

As we consider how to equip future physicians with competencies in critically evaluating research information, the research self-efficacy construct provides a useful framework. If we accept the premise that research training occurs along a continuum that ideally begins with exposure of pre-professional students to the language and culture of research, and extends through post-doctoral training to the independent conduct of multi-center clinical trials, a multi-tiered competency model can be useful to differentiate among basic, intermediate, and advanced research proficiencies. Thus Tier I research competencies are foundational and may require different behavioral domains than those needed to perform research tasks at Tiers II and III.

There is limited information available on strategies for integrating biostatistics and epidemiology into the medical school curriculum, and most research education methods continue to gravitate toward stand-alone traditional graduate courses. Training in the conduct of research during medical school probably should remain separate from the core medical curriculum, but research education for Tier I competencies may be relatively easily incorporated into the curriculum, thus exposing a greater number of students to the language and importance of research in medicine.

## Competing interests

JL is the editor in chief of the journal and removed himself from the review process for this paper.

The other authors have no potential financial conflicts or conflicts of interest.

## Authors' contributions

dAC provided the leadership to the project, developed and administered the questionnaire, analyzed the results, convened the team of authors, wrote the article. BD provided expert input to the content of the questionnaire and to the interpretation of the results. SB provided biostatistics support in the analysis of the data. LB consulted in the development of the questionnaire and provided substantive input to the manuscript in research self-efficacy and interpretation of the *Confidence *factors. JL provided substantive input to the development of the questionnaire and the analysis of the results. AP provided interpretive input to the factor analysis and to the conclusions. RJB contributed to the content of the questionnaire, and substantive input to the content of the article.

All authors read and approved the final manuscript.

## Authors' informations

dAC is the Principal Investigator on the NIH R25 and the OHF Grants that support this initiative for osteopathic medical students in their research education. BD is a collaborator in the development of the curriculum. SB is the research analyst for the project. LB is an expert consultant for the project. JL is a co-investigator in the NIH R25 grant project. AP is a member of the Research Education Committee. RJB is an external partnering consultant.

## References

[B1] Pearson NJ, Chesney MA (2007). The CAM Education Program of the National Center for Complementary and Alternative Medicine: An Overview. Academic Medicine.

[B2] Bandura A (1986). Social foundations of thought and action.

[B3] Segal S, Lloyd T, Houts PS, Stillman PL, Jungas RL, Greer RB (1990). The association between students' research involvement in medical school and their postgraduate medical activities. Academic Medicine.

[B4] Gelso CJ, Mallinckrodt B, Judge AB (1996). Research Training Environment, Attitudes Toward Research, and Research Self-Efficacy: The Revised Research Training Environment Scale. The Counseling psychologist.

[B5] Mullikin E, Bakken L, Betz N (2007). Assessing Research Self-Efficacy in Physician-Scientists: The Clinical Research APPraisal Inventory. Journal of Career Assessment.

[B6] Bieschke KJ, Bishop RM, Garcia VL (1996). The Utility of the Research Self-Efficacy Scale. Journal Of Career Assessment.

[B7] Phillips JC, Russell RK (1994). Research self-efficacy, the research training environment, and research productivity among graduate students in counseling psychology. The Counseling Psychologist.

[B8] Forester M, Kahn JH, Hesson-McInnis MS (2004). Factor Structures of Three Measures of Research Self-Efficacy. Journal Of Career Assessment.

[B9] Betz N (2007). Career Self-Efficacy: Exemplary Recent Research and Emerging Directions. Journal of Career Assessment.

[B10] Bakken LL, Byars-Winston A, Wang MF (2006). Viewing clinical research career development through the lens of social cognitive career theory. Adv Health Sci Educ Theory Pract.

[B11] Bieschke KJ (2006). Research Self-Efficacy Beliefs and Research Outcome Expectations: Implications for Developing Scientifically Minded Psychologists. Journal Of Career Assessment.

[B12] Freeman JV, Collier S, Staniforth D, Smith KJ (2008). Innovations in curriculum design: a multi-disciplinary approach to teaching statistics to undergraduate medical students. BMC Med Educ.

[B13] Motulsky H (1995). Intuitive Biostatistics.

[B14] Cashin S, Elmore P (2005). The Survey of Attitudes Toward Statistics Scale: A Construct Validity Study. Educational and Psychological Measurement.

[B15] Wise SL (1985). The development and validation of a scale measuring attitudes toward statistics. Educational and Psychological Measurement.

[B16] Schau C, Stevens J, Dauphinee TL, Del Vecchio A (1995). The Development and Validation of the Survey of Attitudes Toward Statistics. Educational and psychological measurement.

[B17] West CP, Ficalora RD (2007). Clinician attitudes toward biostatistics. Mayo Clin Proc.

[B18] Windish DM, Huot SJ, Green ML (2007). Medicine residents' understanding of the biostatistics and results in the medical literature. JAMA.

[B19] Jenicek M (2006). The hard art of soft science: Evidence-Based Medicine, Reasoned Medicine or both?. J Eval Clin Pract.

[B20] Ahn J, Watt CD, Man LX, Greeley SA, Shea JA (2007). Educating future leaders of medical research: analysis of student opinions and goals from the MD-PhD SAGE (Students' Attitudes, Goals, and Education) survey. Acad Med.

[B21] Bakken LL (2002). An evaluation plan to assess the process and outcomes of a learner-centered training program for clinical research. Med Teach.

[B22] Moher D, Schulz KF, Altman DG (2001). The CONSORT statement: revised recommendations for improving the quality of reports of parallel-group randomized trials. Ann Intern Med.

[B23] Altman DG, Schulz KF, Moher D, Egger M, Davidoff F, Elbourne D, Gøtzsche PC, Lang T (2001). The revised CONSORT statement for reporting randomized trials: explanation and elaboration. Ann Intern Med.

